# Shear Bond Strength of a Light-Cured and a Dual-Cured Universal Adhesive to Primary and Permanent Dentin: An In Vitro Study

**DOI:** 10.3390/biomimetics11050358

**Published:** 2026-05-21

**Authors:** Ektoras Fousekis, Aristidis Arhakis, Konstantinos Arapostathis, Petros Mourouzis, Kosmas Tolidis, Dimitrios Dionysopoulos

**Affiliations:** 1 Department of Pediatric Dentistry, School of Dentistry, Faculty of Health Sciences, Aristotle University of Thessaloniki, 54124 Thessaloniki, Greece; ekt.foussekis@gmail.com (E.F.); arhakis@dent.auth.gr (A.A.); koarap@dent.auth.gr (K.A.); 2 Department of Operative Dentistry, School of Dentistry, Faculty of Health Sciences, Aristotle University of Thessaloniki, 54124 Thessaloniki, Greece; pmourouzis@dent.auth.gr (P.M.); ktolidis@dent.auth.gr (K.T.)

**Keywords:** dual-cured, light-cured, primary tooth, permanent tooth, shear bond strength, universal adhesive

## Abstract

The aim of the study was to evaluate the immediate and aged shear bond strength (SBS) of a dual-cured and a light-cured universal adhesive to primary and permanent dentin. Twenty caries-free primary molars and twenty permanent third molars were selected and sectioned to expose mid-coronal dentin. Specimens were assigned to four groups (*n* = 10) according to dentition type and adhesive agent used: Group 1—primary teeth/Scotchbond Universal; Group 2—permanent teeth/Scotchbond Universal; Group 3—primary teeth/Futurabond U; and Group 4—permanent teeth/Futurabond U. Two resin micro-rods were fabricated and bonded with the same adhesive on each specimen. The SBS test was applied after 24 h (first micro-rod) and after thermocycling (second micro-rod). Failure modes were assessed under an optical microscope and scanning electron microscopy. Shear bond strength was significantly higher in permanent teeth than in primary teeth (*p* < 0.001). No statistically significant differences were found between the two adhesives (*p* = 0.194). Adhesive failures predominated across all groups. Both adhesives exhibited comparable SBS to primary and permanent dentin. These findings support the suitability of both universal adhesives for the restoration of primary and permanent teeth, though long-term clinical studies are warranted.

## 1. Introduction

From a biomimetic perspective, modern adhesive dentistry aims to replicate the structural and functional characteristics of the natural dentin–enamel complex by establishing a stable, durable, and stress-distributing interface between restorative materials and dental tissues. Universal adhesives, which are designed to interact with both hydroxyapatite and collagen-rich dentin, represent an important step toward this goal by combining micromechanical interlocking with chemical bonding mechanisms that partially mimic natural mineral–organic interactions. In this context, evaluating the performance of these adhesives in both primary and permanent dentition is clinically relevant, as differences in dentin morphology, mineral content, and tubular structure may influence the ability of adhesive systems to achieve biomimetic integration and long-term interfacial stability. Therefore, understanding how universal adhesives behave under immediate and aged conditions contributes to the broader objective of optimizing biomimetic restorative strategies across different dental substrates.

Adhesive systems are essential in minimally invasive restorative dentistry, allowing preservation of sound tooth structure and effective bonding of resin-based materials [1]. The two main categories of dental adhesives were the etch-and-rinse and the self-etch systems. With each new generation, manufacturers have aimed to reduce the number of bottles and procedural steps, as well as to improve chemical formulations to achieve stronger bonding [2].

Bonding to enamel is achieved mainly through micromechanical interlocking, after acid etching creates surface irregularities that allow adhesive resin infiltration [3]. Bonding to dentin is more complex, since dentin is a moist and heterogeneous substrate with different morphological features, including dentinal tubules and collagen. In total-etch technique, phosphoric acid removes the smear layer and demineralizes the superficial dentin, allowing resin monomers to infiltrate the exposed collagen network and form a hybrid layer. In self-etch systems, acidic monomers interact with and partially dissolve the smear layer without completely removing it, resulting in a shallower demineralization pattern and a structurally different adhesive interface [4]. Consequently, the major difference in self-etch adhesives is their capacity to form primary bonds with the partially demineralized substrate.

Universal adhesives have been introduced to simplify bonding procedures and offer flexibility in clinical application, yet their performance may vary depending on the dental substrate and aging conditions. They can be applied using total-etch, selective-etch or self-etch technique and have the capacity to bond to enamel, dentin, ceramics, composites and metal substrates [5]. As a result, they are often referred to as “multi-mode” adhesives. Scotchbond Universal adhesive is among the most widely investigated universal adhesives and is frequently used as a reference material in bonding studies [6]. Among the universal adhesives currently available, Futurabond U (Voco, Anton-Flettner-Straße 1-3, 27472 Cuxhaven, Germany) is a dual-cured nano-reinforced material developed for simplified clinical use across different restorative substrates [7].

Although bonding to permanent enamel and dentin has been extensively investigated, considerably less evidence is available regarding adhesion to primary teeth, especially using dual-cured universal adhesive systems. Structural and compositional differences between primary and permanent dentin may influence the bonding performance of adhesive systems [8]. The aprismatic enamel layer is more pronounced in primary teeth. The dental tissues of primary teeth are also thinner and have a lower concentration of calcium and phosphorous than permanent teeth. Furthermore, primary dentin has a higher tubule density and reduced intertubular dentin available for adhesion [8]. In clinical practice, adhesive systems are often used in primary dentition based on knowledge derived from permanent teeth, while specific protocols for their use in primary teeth remain less clearly established [9]. In addition, although adhesive systems have been significantly improved over the years, the bonding interface continues to be the most vulnerable point of the composite restoration. Hydrolytic and enzymatic degradation, resulting from water absorption and proteolytic activity from saliva and dentin, may compromise the adhesive interface over time [5,10].

Thus, the aim of this in vitro study was to compare the immediate and aged shear bond strength (SBS) of two universal adhesives, a light-cured and a dual-cured, to primary and permanent dentin. The selection of the two adhesives was intended to allow comparison between different polymerization strategies commonly available in clinical adhesive systems. Although dual-cured adhesives are more frequently associated with dual-cured restorative materials, they may also be used with light-cured materials and therefore remain clinically relevant. Including both systems enabled evaluation of whether the curing mode of the adhesive could influence the outcomes investigated in this study.

The following three null hypotheses were set: (1) there would be no difference in SBS between the two universal adhesives; (2) there would be no difference in SBS between primary and permanent teeth; and (3) thermocycling would not significantly affect the SBS of the universal adhesives.

## 2. Materials and Methods

### 2.1. Preparation of the Specimens

This in vitro study was approved by the Ethics Committee of the School of Dentistry, Aristotle University of Thessaloniki, Thessaloniki, Greece (Approval No: 23/09-07-2024). A total of 20 extracted, caries-free, unrestored primary molars and 20 extracted, caries-free, unrestored permanent third molars were selected after obtaining patient consent. Following extraction, the teeth were stored in a 0.5% chloramine-T solution for up to 1 month.

For specimen preparation, teeth were sectioned parallel to the occlusal surface using a diamond disc mounted on a precision cutting machine (Isomet Buehler, Lake Bluff, IL, USA) under copious water spray in order to expose mid-coronal dentin. The sectioned teeth were then embedded in self-curing acrylic resin (Kallocryl, SPEIKO, Münster, Germany) within silicone molds and allowed to set. The dentin surfaces were polished using a polishing machine (Jean Wirtz, TG 250, Dusseldorf, Germany) with 600-grit silicon carbide paper (Struers, Copenhagen, Denmark) to create a standardized smear layer, similar to that produced during clinical instrumentation [11].

### 2.2. Experimental Groups of the Study

The tooth specimens were then randomly allocated to four groups (n = 10) according to dentition type and adhesive agent used, as follows: Group 1—primary teeth/Scotchbond Universal; Group 2—permanent teeth/Scotchbond Universal; Group 3—primary teeth/Futurabond U; and Group 4—permanent teeth/Futurabond U.

### 2.3. Bonding Procedures

On dentin surfaces, two specimens of the two tested universal adhesives (Table 1), Scotchbond Universal (3M ESPE, St. Paul, MN, USA) and Futurabond U (VOCO GmbH, Cuxhaven, Germany), were bonded. Each adhesive was applied with a micro-applicator for 10 s, gently air-dried for 5 s and light-cured for 10 s with an LED light-curing unit (Elipar S10, 3M ESPE, St. Paul, MN, USA) operating at 1200 mW/cm^2^ with standard curing mode. Separate etching of dentin prior to bonding was not implemented.

Subsequently, micro-rods were fabricated using a methacrylate-based flowable resin composite (GrandioSO Heavy Flow, HVM, Voco, Germany) (Table 1) and polyethylene Tygon tubes (Tygon^®^ S3 E−3603 Tubes International Sp. Poznan, Poland, internal diameter: 1.5 mm; height 3 mm). On each dentin specimen, two micro-rods were bonded using one of the two tested universal adhesives. The resin composite was light-cured for 20 s from each side using the same LED curing unit as described above. All specimens were then stored in distilled water at 37 °C for 24 h.

### 2.4. Shear Bond Strength Test

**Figure 1:**
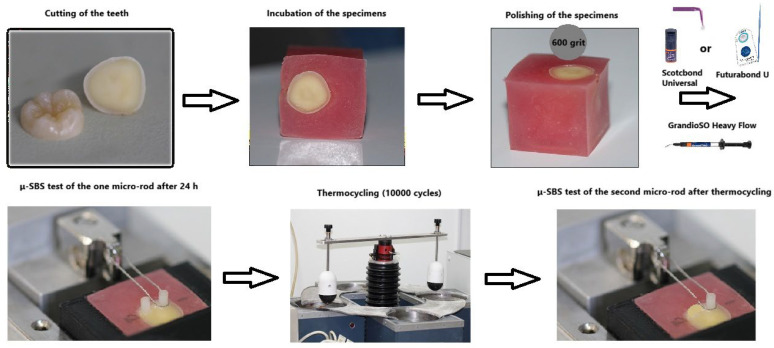
The experimental design of the study.

No pretesting failures occurred before the SBS test. After 24 h of water storage at 37 °C, one of the two bonded micro-rods was mounted in the lower fixture of a shear testing machine (Odeme Dental Research, Luzerna, SC, Brazil) for SBS evaluation. An orthodontic stainless steel wire (cyclic cross-section) of 0.2 mm in diameter (Standard stainless steel round wire, American Orthodontics, Sheboygan, WI, USA) was looped around each resin micro-rod and placed as close as possible to the resin–dentin interface. A shear load was then applied at a crosshead speed of 0.5 mm/min until failure occurred. Bond strength values (MPa) were calculated by dividing the load at failure (N) by the bonded area (~1.77 mm^2^). The remaining micro-rods were subjected to 10,000 thermal cycles between 5 °C and 55 °C with a dwell time of 10 s and a transfer time of 5 s, to simulate intraoral temperature changes [12]. Following thermocycling, SBS test was performed using the same testing protocol and the same shear testing machine as described before. The workflow of the experimental part of the study is illustrated in Figure 1.

### 2.5. Failure Mode Analysis

Fractured specimens were inspected under an optical microscope (SJ Optic GmbH, Königsbach-Stein, Germany) at ×15 magnification to assess failure mode. Failure modes were classified as: A: “adhesive” (plain interfacial failure), B: “resin cohesive” (all bonding surface covered with resin), C: “mixed” (part of resin covering the bonding surface, the extent of which can be easily measured with image processing) and D: “dentin cohesive” (evidence of dentin failure regardless of the extent of resin residues left). The assessment was performed by a single examiner blinded to dentition type, aging condition, and adhesive type.

### 2.6. Scanning Electron Microscopy Analysis

Following the SBS test, the debonded tooth surfaces were analyzed to determine failure modes using scanning electron microscopy (SEM; JEOL Ltd., JSM-840, Tokyo, Japan) at magnifications up to ×40. Specimens were mounted on aluminum stubs and sputter-coated with a carbon layer approximately 200 Å thick under low-vacuum conditions in a vacuum evaporator. SEM observations were conducted at an accelerating voltage of 20 kV.

### 2.7. Statistical Analysis

An a priori sample-size calculation was performed using G*Power 3.1. The analysis was based on the F tests family and the procedure “ANOVA: Fixed effects, special, main effects and interactions”, considering the planned 2 × 2 × 2 factorial experimental structure of the study. A two-sided significance level of α = 0.05, a desired statistical power of 1 − β = 0.80, and balanced allocation across experimental conditions were specified. The effect-size metric was Cohen’s f, defined a priori on the basis of external information from the relevant literature and a clinically meaningful expected difference. The calculation indicated a planned total of 80 SBS observations, corresponding to 40 tooth specimens, with 10 specimens allocated to each Adhesive × Dentition combination and two aging-related SBS measurements obtained per tooth specimen.

Shear bond strength data were analyzed within a 2 × 2 × 2 mixed factorial design, with adhesive system and dentition type as between-specimen factors and aging condition as a within-specimen factor, because 24 h and thermocycled measurements were obtained from micro-rods bonded to the same tooth specimen. Descriptive data are presented as mean ± standard deviation. Normality was assessed using Shapiro–Wilk tests, and homogeneity of variance using the median-centered Levene’s test. Because variance heterogeneity was detected and diagnostic assessment of a preliminary mixed-effects model indicated non-normal residuals, SBS data were analyzed using a rank-based nonparametric factorial repeated-measures approach for an F2-LD-F1 design. The model tested the main effects of dentition, adhesive, and aging, together with all two-way and three-way interactions. Modified ANOVA-type statistics with Box approximation were reported for between-specimen effects and ANOVA-type statistics for aging and aging-related interactions. As each factor had only two levels and no interaction term was statistically significant, no post hoc multiple-comparison procedures were required. Failure-mode distributions were compared among experimental groups using Fisher’s exact test with Monte Carlo simulated *p*-values based on 100,000 replicates. All tests were two-sided, with α = 0.05. Analyses were performed in R version 4.5.1.

## 3. Results

### 3.1. Shear Bond Strength Outcomes

Descriptive SBS values for the eight experimental conditions are presented in Table 2. Across the experimental groups, mean SBS values ranged from 13.27 ± 0.98 MPa for primary dentin bonded with Futurabond U without thermocycling to 16.75 ± 2.51 MPa for permanent dentin bonded with Scotchbond Universal without thermocycling. The nonparametric mixed factorial repeated-measures analysis revealed a statistically significant main effect of dentition type on SBS. Permanent dentin exhibited higher bond-strength values than primary dentin, as reflected by the corresponding relative treatment effects (RTE = 0.653 for permanent dentin and RTE = 0.348 for primary dentin; modified ANOVA-type statistic with Box approximation = 28.327, df(_1) = 1, df(_2) = 33.342, *p* < 0.0001). No statistically significant main effect of adhesive system was detected (statistic = 1.756, df(_1) = 1, df(_2) = 33.342, *p* = 0.194). Scotchbond Universal showed numerically higher SBS values than Futurabond U in several experimental conditions, but this difference was not statistically significant overall. Likewise, aging condition did not exert a statistically significant effect on SBS (ANOVA-type statistic = 0.789, df = 1, *p* = 0.374). None of the interaction terms reached statistical significance. Specifically, the Dentition × Adhesive interaction was non-significant (statistic = 1.670, df(_1) = 1, df(_2) = 33.342, *p* = 0.205), as were the Dentition × Aging (statistic = 3.395, df = 1, *p* = 0.065), Adhesive × Aging (statistic = 0.004, df = 1, *p* = 0.952), and Dentition × Adhesive × Aging interactions (statistic = 0.137, df = 1, *p* = 0.712). Thus, the higher SBS observed in permanent dentin was consistent across adhesive systems and aging conditions.

### 3.2. Failure Mode Analysis Outcomes

**Figure 2:**
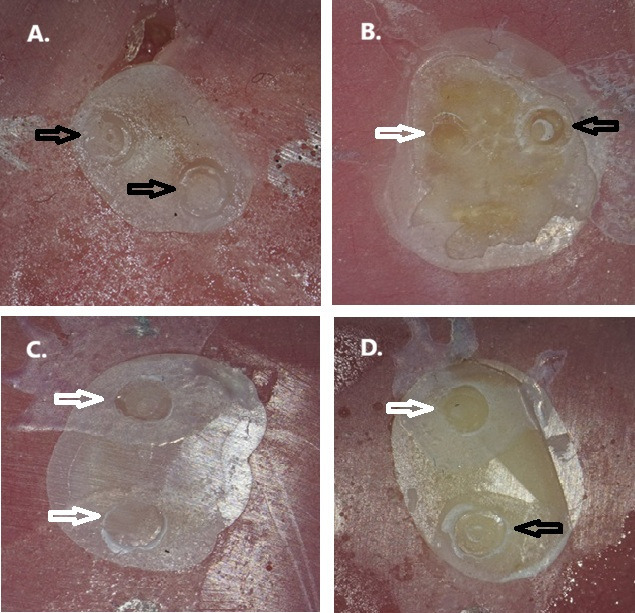
Representative images of specimens using an optical microscope (×15 magnification) of each experimental group of the study after SBS test showing the failure type. (**A**) primary tooth with Scotchbond Universal; (**B**) permanent tooth with Scotchbond Universal; (**C**) primary tooth with Futurabond U; and (**D**) permanent tooth with Futurabond U. Black arrows indicate mixed failure mode and white arrows indicate adhesive failure mode.

**Figure 3:**
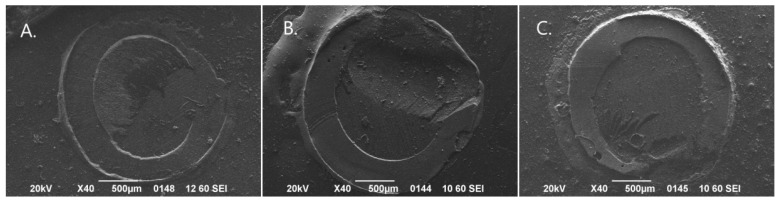
Representative SEM images (×40 magnification) of failure modes. (**A**) mixed failure at the adhesive/restorative material interface, (**B**) mixed failure at the adhesive/dentin interface, and (**C**) adhesive failure.

The distribution of failure modes across the experimental groups is shown in Table 3. Overall, adhesive failures predominated, accounting for 74 of 80 specimens (92.5%), while mixed failures were observed in 6 specimens (7.5%). No cohesive failures were recorded. Adhesive failures ranged from 80% to 100% across individual experimental groups. Mixed failures were observed only in selected groups, namely, permanent dentin bonded with Scotchbond Universal before and after thermocycling, primary dentin bonded with Futurabond U before thermocycling, and permanent dentin bonded with Futurabond U before thermocycling. The global comparison of failure-mode distributions among the eight experimental groups revealed no statistically significant difference (Fisher’s exact test with Monte Carlo simulated *p*-value = 0.487). Representative optical microscopy images of the observed failure patterns are presented in Figure 2, while SEM findings supporting failure-mode characterization are shown in Figure 3.

## 4. Discussion

In recent years, the demand for tooth-colored restorations has increased in the field of pediatric dentistry. Additionally, advances in minimally invasive dentistry have led to a growing demand among dental professionals for adhesive materials with simplified bonding protocols that reduce the number of clinical steps required during restorative procedures [13]. The growing demand for such materials has driven the development of universal adhesives, which can be used either in self-etch (SE) or etch-and-rinse (ER) mode. Because these adhesives are relatively new, information on their long-term clinical performance is still limited.

In a recent systematic review, Triani et al. [13] evaluated the efficiency of universal adhesive bonding to enamel and dentin of permanent teeth. They reported that ER approach showed better results for enamel, while the adhesive strategy did not significantly affect the bond strength to dentin [13]. However, they noted that when universal adhesives are used in SE mode, mild or intermediate acidic monomers partially demineralize the smear layer and underlying tooth structure without excessively exposing the collagen fiber network, thereby preventing its collapse. Rosa et al. [3] in a similar systematic review indicated that selective etching of enamel could be considered the best strategy for optimizing universal adhesive bond strength. Regarding primary teeth, Fröhlich et al. [14] reported that both SE and ER strategies did not influence the primary dentin immediate bond strength [14]. Thus, for the present study, the SE approach was adopted.

Clinicians have utilized multiple tests over the years to assess adhesion, including tensile, micro-tensile, shear, and micro-shear tests. Shear bond strength tests are the most commonly applied, largely due to their simplicity. However, conventional macro tests can lead to non-uniform stress distribution, as internal defects have a greater impact on larger bonding areas [15]. Micro tests have been developed to address these limitations by reducing variability, increasing cohesive failures, and allowing multiple measurements from a single specimen. A notable disadvantage, however, is their higher technique sensitivity [16].

The micro-shear bond strength test, in particular, measures small bonding areas and allows for a greater number of samples to be obtained from a single tooth. It is also less technique-sensitive than the micro-tensile test [17]. The test can be conducted using either a chisel or a wire loop [18]. While a chisel tends to create higher stress concentrations at the point of force application, the wire loop distributes forces more evenly across the interface [19]. Therefore, in our study, the test was performed using a wire loop.

For this particular study, the two universal adhesives assessed were Scotchbond Universal (light-cured) and Futurabond U (dual-cured). Dual-cured universal adhesives, such as Futurabond U, exhibit more reliable polymerization in deep or inaccessible areas; they are not dependent on restoration translucency or curing angle, and can be applied in thinner layers, providing improved seating of indirect restorations [20]. On the other hand, previous studies have reported reduced bond strength if they are not light-activated [20]. Moreover, they present more complex handling because they often require mixing components, leading to a higher risk of error, as well as contain chemical initiators (e.g., sulphinate, vanadium or copper salts) that may cause more discoloration over time compared to purely light-cured systems [21].

A highly filled flowable composite material was selected for the fabrication of resin micro-rods (GrandioSO Heavy Flow), as adequate compaction of a conventional composite within the Tygon tubes would be technically challenging. According to the manufacturer, these highly filled flowable composites exhibit exceptionally high filler loading (80–83 wt%) and demonstrate reduced polymerization shrinkage (2.96%) [22]. With respect to their mechanical performance, these highly filled flowable composites approximate the properties of conventional restorative composites more closely than traditional flowable materials.

Scotchbond Universal, compared to Futurabond U, did not present statistically significant difference in immediate bond strength. The absence of a statistically significant difference in the current study suggests that both adhesives achieve comparable micromechanical interlocking and chemical interaction with dentin. In a similar study, Ahmed et al. [23] reported that Futurabond U had inferior SBS values as compared to other tested universal adhesives. They suggested that the higher solvent concentration (25–50 wt%) in Futurabond U could lead to more residual solvent being retained within the hybrid and adhesive layers. On the other hand, considering that both adhesives contain ethanol and water as solvents, the major difference between the two is that the light-cured is separately irradiated, providing a well-defined film where the restorative is placed onto. The dual-cured does not set prior to restorative application, and on flat surfaces, it may be easily displaced. As a result, it can be postulated that it is not an issue of solvents, since the amount in the initial material is strongly reduced upon air-drying.

Nevertheless, dental professionals are concerned not only with the immediate bond strength of adhesive materials but also with their long-term clinical performance. For this reason, in vitro aging methods are considered essential in any laboratory adhesion study, as they provide insight into the durability of adhesive systems and help predict the effects of bonding degradation over time [24]. Different aging methods—including water storage, thermocycling, NaOCl immersion, or their combinations—are used to simulate long-term adhesive performance. Among these, thermocycling appears to be able to produce more consistent and predictable results [25].

After thermocycling, the difference in bond strength between Scotchbond Universal and Futurabond U, both in primary and permanent dentin, was also not statistically significant. These findings indicated that compositional differences between the two adhesives may have a small effect on their initial performance, but thermocycling tends to level out these differences as both interfaces undergo similar degradation processes. Therefore, the first null hypothesis, stating that the two universal adhesives would exhibit no significant differences in SBS values, was accepted.

Higher bond strength values were observed in permanent dentin than in primary dentin, regardless of the adhesive used or whether thermocycling was performed. These findings are consistent with those reported by Ghajari et al. [26,27], who suggested that microstructural features, such as fewer and smaller dentinal tubules with lower permeability, increased reactivity, and reduced calcium and phosphate in intertubular and peritubular dentin, likely underlie these results [26]. The hybrid layer in primary teeth is considerably thicker than in permanent teeth, suggesting that primary dentin is more reactive to acid conditioning. To improve adhesion, some authors recommended shortening the etching time, which can lead to a more uniform hybrid layer and stronger bonds [27]. Therefore, the second null hypothesis, which assumed no significant differences in SBS between primary and permanent teeth, was rejected.

Thermocycling resulted in a slight numerical reduction in bond strength for both adhesives; however, this difference was not statistically significant. This finding may be attributed to the moderate aging effect of the applied thermocycling protocol. It is possible that increasing the number of thermal cycles could yield a statistically significant difference. Additionally, the dwell time used (10 s) was relatively low compared to previous studies. It has been documented that the dwell time for such applications should range between 20 and 60 s (preferably 40–60 s) to provide enough time for the core material to reach the specific temperature [28]. Probably, this was the main reason why no effects were registered after thermocycling in the present study. Nevertheless, in the aforementioned study by Eliasson and Dahl [28], the number of thermal cycles was half (5000 cycles) of that applied in the current study. In the present study, the 10 s dwell time was adopted by earlier reports [29,30]. It is still questionable which the optimal thermocycling protocol for composite bonding studies is, and as a result, more evidence is needed to conclude on a more broadly accepted protocol for artificial aging.

Another possible explanation for these results is the presence of functional monomers capable of chemical interaction with dentin, and the mild self-etching behavior of both universal adhesives, which has been associated with improved resistance to hydrolytic degradation. Scotchbond Universal features 2-propenoic acid, 2-methyl-, reaction products with 1,10-decanediol and phosphorous oxide, which are chemically related to 10-methacryloyloxydecyl dihydrogen phosphate (10-MDP), whereas Futurabond U’s functional monomer is described by the manufacturer simply as a phosphate monomethacrylate. Most universal adhesives in the market contain 10-MDP. This organic chemical compound creates a strong interaction with hard dental tissues by inducing the surface dissolution of hydroxyapatite and the subsequent formation of MDP–calcium salts. The stability of this calcium salt contributes to the strong bonding affinity of 10-MDP to both enamel and dentin [31]. Consequently, the third null hypothesis that thermocycling would not alter the shear bond strength of both adhesives was also accepted.

The lack of pretesting failures suggests that the bonding procedures and specimen preparation were carried out consistently, and that all specimens possessed sufficient initial bond strength to endure handling and testing [32]. It has been reported that failure mode is closely related to bond strength, with weaker adhesive systems exhibiting predominantly adhesive failures due to limited resin penetration, whereas cohesive failures are typically associated with stronger bonding performance [33]. The predominance of adhesive failures and the absence of cohesive failures observed in the present study can be attributed to several methodological and substrate-related factors. Micro-shear bond strength tests are known to produce mainly adhesive failures due to the small bonding area and stress concentration at the adhesive interface, which promotes interfacial debonding rather than cohesive fracture [19]. Furthermore, the use of universal adhesives in self-etch mode, without prior phosphoric acid etching of dentin, may have contributed to weaker interfacial bonding, as these systems rely on milder etching capacity, resulting in less pronounced demineralization and shallower resin tag formation. In similar in vitro studies, Ghatjari et al. [8] and Can-Karabulut et al. [27] found that in self-etch mode, universal adhesives exhibited mostly adhesive or mixed-type failures. This is consistent with the results of the current study.

Several limitations should be considered when interpreting the results of the present study. First, similarly to all in vitro investigations, the findings may not fully reflect the complex conditions of the oral environment [34] ]. Furthermore, a larger sample size for both primary and permanent teeth or more aggressive aging protocols with longer dwell times could have revealed more statistically significant results. Moreover, the inclusion of additional aging protocols, such as mechanical loading and long-term water storage, would have further enhanced the clinical relevance of the study. In addition, the current study evaluated the bond strength of the universal adhesives only in self-etch mode. Nevertheless, the comparisons between the experimental groups remain valid and could provide useful insights for clinical decision-making. Dual-cured universal adhesives should be applied in cases where light-curing is problematic and difficult to achieve sufficiently, such as in cases of deep cavities or inaccessible areas in children or adults.

## 5. Conclusions

Within the limitations of the present in vitro study, both universal adhesives, Scotchbond Universal and Futurabond U, demonstrated comparable shear bond strength to primary and permanent dentin. Permanent dentin consistently showed higher bond strength than primary dentin, possibly due to morphological and chemical differences in their microstructure. Bond strength was not significantly impacted by thermocycling under the applied conditions, indicating that these adhesives have a moderate resistance to short-term hydrolytic degradation. Based on SBS findings of the present study, the use of both universal adhesives for the restoration of primary and permanent teeth is feasible. However, the absence of cohesive failures may also suggest that the interfacial bond remained the weakest region of the bonded assembly, so the interpretation of the results should be more critical and cautious. Additional long-term clinical studies are required to validate their performance and durability in complex oral conditions.

## Figures and Tables

**Table 1:** Technical characteristics of the tested materials.

Material	Manufacturer	Composition
Adper Scotchbond Universal	3M ESPE, St. Paul, MN, USA	2-hydroxyethyl methacrylate, bisphenol A diglycidyl ether dimethacrylate, decamethylene dimethacrylate, ethanol, silane treated silica, water, 2-propenoic acid, 2-methyl-, reaction products with 1,10-decanediol and phosphorus oxide, copolymer of acrylic and itaconic acid, dimethylamino ethyl methacrylate, camphorquinone, dimethylaminobenzoate, 2,6-di-tert-butyl-P-cresol
Futurabond U	VOCO, GmbH, Cuxhaven, Germany	Bisphenol A diglycidyl methacrylate, 2-hydroxyethyl methacrylate, 1,6-hexanediyl bismethacrylate, acidic adhesive monomer, urethane dimethacrylate, catalyst
GrandioSO Heavy Flow	VOCO, GmbH, Cuxhaven, Germany	Organic Matrix: Bis-GMA, UDMA, TEGDMA; Inorganic Fillers: Barium glass, Silica; Initiators: Camphorquinone, DMAEMA; Stabilizers/Modifiers: BHT; Additives: Pigments, viscosity modifiers

BHT: butylated hydroxytoluene; Bis-GMA: bisphenol A-glycidyl methacrylate; DMAEMA: 2-(Dimethyl amino)ethylmethacrylate; TEGDMA: triethylene glucol dimethacrylate; UDMA: urethane dimethacrylate.

**Table 2:** Mean shear bond strength (MPa) and corresponding standard deviation (SD) of the experimental group of the study (n = 10).

Groups	Adhesive	Dentition	After 24 h	After Thermocycling
1	Scotchbond Universal	Primary	13.97 ± 1.33 ^Aa^	14.42 ± 1.65 ^ABa^
2	Scotchbond Universal	Permanent	16.75 ± 2.51 ^Ba^	15.24 ± 2.04 ^Ba^
3	Futurabond U	Primary	13.27 ± 0.98 ^Aa^	13.46 ± 1.11 ^Aa^
4	Futurabond U	Permanent	15.84 ± 1.10 ^Ba^	15.50 ± 2.22 ^Ba^

Same uppercase superscripts in columns indicate no statistically significant differences among the experimental groups (*p* > 0.05). Same lowercase superscripts in rows indicate no statistically significant differences between non-thermocycled and thermocycled groups (*p* > 0.05).

**Table 3:** Failure type distribution in the experimental groups of the study.

Dentition	Adhesive System	Thermocycling	Adhesive (%)	Mixed (%)	Resin Cohesive (%)	Dentin Cohesive (%)
Permanent	Scotchbond Universal	No	8 (80%)	2 (20%)	0 (0%)	0 (0%)
Permanent	Futurabond U	No	9 (90%)	1 (10%)	0 (0%)	0 (0%)
Primary	Scotchbond Universal	No	10 (100%)	0 (0%)	0 (0%)	0 (0%)
Primary	Futurabond U	No	9 (90%)	1 (10%)	0 (0%)	0 (0%)
Permanent	Scotchbond Universal	Yes	8 (80%)	2 (20%)	0 (0%)	0 (0%)
Permanent	Futurabond U	Yes	10 (100%)	0 (0%)	0 (0%)	0 (0%)
Primary	Scotchbond Universal	Yes	10 (100%)	0 0%	0 (0%)	0 (0%)
Primary	Futurabond U	Yes	10 (100%)	0 (0%)	0 (0%)	0 (0%)

## Data Availability

The data presented in this study are available upon request from the corresponding author. The data are not publicly available due to privacy.
